# Failure of a numerical quality assessment scale to identify potential risk of bias in a systematic review: a comparison study

**DOI:** 10.1186/s13104-015-1181-1

**Published:** 2015-06-06

**Authors:** Seán R O’Connor, Mark A Tully, Brigid Ryan, Judy M Bradley, George D Baxter, Suzanne M McDonough

**Affiliations:** Centre for Public Health, Queen’s University Belfast, Belfast, UK; UKCRC Centre of Excellence for Public Health (Northern Ireland), Belfast, UK; Centre for Health, Activity and Rehabilitation Research, University of Otago, Dunedin, New Zealand; Centre for Health and Rehabilitation Technologies, Institute of Nursing and Health Research, School of Health Sciences, University of Ulster, Belfast, UK

**Keywords:** Quality assessment, Risk of bias, Systematic review methods

## Abstract

**Background:**

Assessing methodological quality of primary studies is an essential component of systematic reviews. Following a systematic review which used a domain based system [United States Preventative Services Task Force (USPSTF)] to assess methodological quality, a commonly used numerical rating scale (Downs and Black) was also used to evaluate the included studies and comparisons were made between quality ratings assigned using the two different methods. Both tools were used to assess the 20 randomized and quasi-randomized controlled trials examining an exercise intervention for chronic musculoskeletal pain which were included in the review. Inter-rater reliability and levels of agreement were determined using intraclass correlation coefficients (ICC). Influence of quality on pooled effect size was examined by calculating the between group standardized mean difference (SMD).

**Results:**

Inter-rater reliability indicated at least substantial levels of agreement for the USPSTF system (ICC 0.85; 95% CI 0.66, 0.94) and Downs and Black scale (ICC 0.94; 95% CI 0.84, 0.97). Overall level of agreement between tools (ICC 0.80; 95% CI 0.57, 0.92) was also good. However, the USPSTF system identified a number of studies (n = 3/20) as “poor” due to potential risks of bias. Analysis revealed substantially greater pooled effect sizes in these studies (SMD −2.51; 95% CI −4.21, −0.82) compared to those rated as “fair” (SMD −0.45; 95% CI −0.65, −0.25) or “good” (SMD −0.38; 95% CI −0.69, −0.08).

**Conclusions:**

In this example, use of a numerical rating scale failed to identify studies at increased risk of bias, and could have potentially led to imprecise estimates of treatment effect. Although based on a small number of included studies within an existing systematic review, we found the domain based system provided a more structured framework by which qualitative decisions concerning overall quality could be made, and was useful for detecting potential sources of bias in the available evidence.

**Electronic supplementary material:**

The online version of this article (doi:10.1186/s13104-015-1181-1) contains supplementary material, which is available to authorized users.

## Background

Systematic reviews are used to synthesize research evidence relating to the effectiveness of an intervention [1]. Conclusions of high quality reviews provide a basis on which clinicians and researchers can make evidence-based decisions and recommendations. Accurately assessing methodological quality of included studies is therefore essential. Quality is a multidimensional concept representing the extent to which study design can minimise systematic and non-systematic bias, as well as inferential error [2, 3].

There are numerous instruments available for assessing quality of evidence and there remains uncertainty over which are the most appropriate to use [4], and how they should be used to interpret results [5, 6]. Use of different assessment methods can result in significant changes to the size and direction of pooled effect sizes [7–9] and it is therefore important to consider the properties of the assessment methods used.

Numerical summary scores may be of limited value in interpreting the results of meta-analyses [10]. However, these scales are widely used in the literature, possibly due to their ease of use. Quality assessment based on non-numerical or domain-based rating systems [11–14] are increasingly used, particularly when also seeking to make treatment recommendations.

The primary aim of this study was to compare these two contrasting methods for assessing methodological quality of randomized and non-randomized studies included within a systematic review [15]. As part of the review the rating system proposed by the United States Preventative Services Task Force (USPSTF) [12, 13] was used to assess methodological quality and to allow for treatment recommendations to be made. We wished to compare this domain based rating system to a numerical scale to determine the potential influence of different approaches on treatment effect size within a review. We selected the rating scale proposed by Downs and Black [16] for comparison as it is one of the most commonly used and well validated numerical rating scales [17].

The study objectives were:

1. To determine the effect of quality ratings on pooled effect size for primary outcome data from the included studies.
2. To determine inter-rater reliability and level of agreement between tools when examining separate components of internal and external validity, as well as overall ratings assigned to each paper.

## Methods

### Details of each quality assessment tool

#### Downs and Black Scale

The Downs and Black Scale consists of 27 questions relating to quality of reporting (ten questions), external validity (three questions), internal validity (bias and confounding) (13 questions), and statistical power (one question) (Additional file 1: Table S1). It has been shown to have high internal consistency for the total score assigned (Kuder–Richardson 20 test: 0.89) as well as all subscales, except external validity (0.54); with reliability of the subscales varying from “good” (bias) to “poor” (external validity) [16]. The original scale provides a total score out of 32 points, with one question in the reporting section carrying a possible two points, and the statistical power question carrying a possible five points. Previous studies have frequently employed a modified version by simplifying the power question and awarding a single point if a study had sufficient power to detect a clinically important effect, where the probability value for a difference being due to chance is <5% [18–20]. The modified version which we employed in this study therefore has a maximum score of 28. Each paper was assigned a grade of “excellent” (24–28 points), “good” (19–23 points), “fair” (14–18 points) or “poor” (<14 points).

### United States Preventative Services Task Force

In rating quality, the USPSTF system assigns individual studies a grade of “good”, “fair”, or “poor” for both internal and external validity. Assessment criteria are not used as rigid rules, but as guidelines with exceptions made if there is adequate justification. In general, a “good” study meets all criteria for that study design; a “fair” study does not meet all criteria but is judged to have no serious flaw that may compromise results; and a “poor” study contains a potentially serious methodological flaw. Criteria for determining a serious flaw are dependent on study design but include lack of adequate randomization or allocation concealment in randomized controlled trials; failure to maintain comparable groups or account for loss to follow-up or lack of similarity between the study population and patients seen in clinical practice [12].

### Quality assessment conducted using both tools

Twenty studies were included as part of an updated systematic review conducted following the “preferred reporting items for systematic reviews and meta-analyses” (PRISMA) [21] guidelines which examined the effects of an exercise intervention for chronic musculoskeletal pain [15] (References for included studies are shown in Additional file 2: Table S2). Each study was assessed independently by two reviewers [GDB, BR] using the Downs and Black scale. Discrepancies were resolved via discussion with a third reviewer [SOC]. The USPSTF was initially used to rate each study by a single reviewer [SOC] and then, as recommended by the USPSTF [12, 13], via consensus decisions made at meetings between review authors [MT, GDB, JB, SM, SOC]. All reviewers had experience of conducting systematic reviews in the area and specific experience of using both measures. Reviewers were not blinded with regards to study authorship, institution, or journal of publication. Prior to assessment reviewers met to establish standardized methods of scoring. Both methods were piloted on a sample of papers examining exercise interventions for an unrelated musculoskeletal condition.

## Analysis

Inter-rater reliability was examined for the separate domains of internal and external validity, as well as for overall quality ratings. Agreement between reviewers before consensus and agreement between tools were determined using the interclass correlation coefficient (ICC) based on a mixed-model, two way analysis of variance (2, k) for absolute agreement and 95% confidence intervals (95% CI). For the purposes of the analysis, when rating quality using the USPSTF system, the number of relevant criteria which were met according to the design of the individual study was used to assign a score out of 11. The Downs and Black scale was scored out of 28. Scores were converted to a percentage (score for paper/total possible score × 100) in order to allow for statistical comparisons to be made between tools.

Criteria used to determine levels of agreement for ICCs were: <0.00 for poor; 0.00–0.20 for fair; 0.21–0.45 for moderate; 0.46–0.75 for substantial and 0.76–1.0 for almost perfect agreement [22]. All analyses were performed using SPSS version 20.0 (SPSS Inc., Chicago, IL, USA). The grading system for the Downs and Black scale was modified to allow comparisons to be made with the USPSTF system by collapsing the “excellent” and “good” ratings together. This meant both tools were used to assign a grade of “good”, “fair” or “poor” to each study. The influence of methodological quality (“poor”, “fair”, or “good”) on pooled effect size for pain data was determined using a random effects model for inverse variance which was used to calculate the standardized mean difference (SMD) and 95% CI [Review Manager (RevMan) (Computer program); Version 5.0] [23].

## Results

Inter-rater reliability for the Downs and Black scale across the separate domains of internal and external validity indicated substantial to almost perfect agreement (ICC = 0.61; 95% CI 0.26, 0.83 and ICC = 0.76; 95% CI = 0.51, 0.90). High levels of agreement were also found for total scores (ICC = 0.94; 95% CI 0.84, 0.97). Scores ranged from 15 to 24/28, with a mean of 18.7 (SD: 2.9).

For the USPSTF system, inter-rater reliability for internal and external validity was also good (ICC = 0.67; 95% CI = 0.33, 0.85 and ICC = 0.84; 95% CI = 0.63, 0.93 respectively). High levels of agreement were also observed for total scores assigned (ICC = 0.85; 95% CI = 0.66, 0.94).

**Figure 1:**
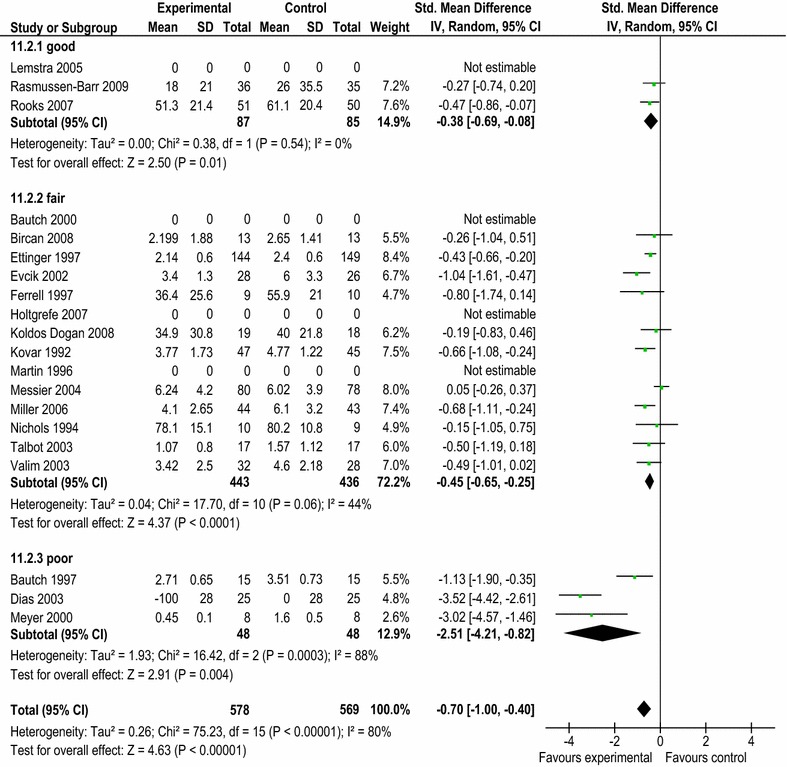
Estimates of treatment effect size for pain data according to quality rating using the United States Preventative Services Task Force (USPSTF) system.

There was at least a substantial level of agreement between the total scores assigned to each paper using both tools (ICC = 0.80; 95% CI = 0.57, 0.92) and overall quality ratings were the same for 14/20 studies (Table 1). However, the USPSTF system identified a small number of studies (n = 3/20) as “poor” which the Downs and Black scale did not. Analysis of pooled effect sizes for pain data revealed substantial differences between these studies compared to those rated as “fair” or “good”, with a SMD (95% CI) of −2.51 (−4.21, −0.82); −0.45 (−0.65, −0.25); and −0.38 (−0.69, −0.08) respectively (Figure 1).

**Table 1:** Comparison of quality ratingsassigned to each paper using the Downs and Black (DB) scale and United States Preventative Services Task Force (USPSTF) system

References	DB (internal validity score/13)	USPSTF (internal validity rating)	DB (external validity score/3)	USPSTF (external validity rating)	DB (total numerical score^b^/28 and rating)	USPSTF (overall rating)
*Bautch et al (1997 )*	*8*	*Poor*	*1*	*Fair*	*18; fair*	*Poor* ^a^
Bautch et al. (2000)	6	Fair	1	Fair	17; fair	Fair
Bircan et al. (2008)	7	Fair	1	Fair	18; fair	Fair
*Dias et al. (2003)*	*8*	*Poor*	*1*	*Fair*	*18; fair*	*Poor* ^a^
*Ettinger et al. (1997)*	*11*	*Good*	*3*	*Fair*	*25; good*	*Fair* ^a^
Evcik et al. (2002)	5	Fair	1	Good	15; fair	Fair
Ferrell et al. (1997)	8	Fair	1	Fair	19; fair	Fair
Holtgrefe et al. (2007)	7	Fair	3	Fair	18; fair	Fair
Koldas Doğan et al. (2008)	7	Fair	0	Fair	17; fair	Fair
*Kovar et al. (1992)*	*9*	*Fair*	*3*	*Fair*	*21; good*	*Fair* ^a^
Lemstra et al. (2005)	11	Good	3	Good	24; good	Good
Martin et al. (1996)	7	Fair	1	Fair	16; fair	Fair
*Messier et al. (2004)*	*11*	*Fair*	*3*	*Fair*	*23; good*	*Fair* ^a^
*Meyer et al. (2000*)	*8*	*Poor*	*0*	*Poor*	*19; fair*	*Poor* ^a^
Miller et al. (2006)	8	Fair	1	Fair	17; fair	Fair
Nichols et al. (1994)	8	Fair	1	Fair	15; fair	Fair
Rasmussen-Barr et al. (2009)	9	Good	2	Good	21; good	Good
Rooks et al. (2007)	10	Good	3	Good	24; good	Good
Talbot et al. (2003)	7	Fair	2	Good	18; fair	Fair
Valim et al. (2003)	7	Fair	1	Poor	19; fair	Fair

Separate scores are given for each section (reporting, internal validity, external validity) and the overall total score. Papers were rated as “Excellent/good”, “fair” or “poor” depending on the numerical score assigned to the paper (Excellent/Good = 20–28; Fair = 15–19; Poor = <14).

^a^Italicized studies indicate where the final grade assigned to the paper differed depending on the quality assessment tool used.

^b^Total possible score for the modified D&B scale = 28; reporting) = 11; internal validity = 13; external validity = 3; power = 1.

## Discussion

### Comparison between tools

This study examined the inter-rater reliability and level of agreement between two different approaches used to assess the methodological quality of randomized and non-randomized studies within a systematic review [15]. Both tools demonstrated good inter-rater reliability across the separate domains of internal and external validity, as well as for the final grade assigned to each paper. Although both tools assigned markedly different weighting to the internal and external validity sections, agreement was also good for the final grades assigned.

While overall analysis indicated a high level of agreement; the domain-based USPSTF system identified a number of the studies (3/20) as “poor” due to potential sources of bias. These studies were found to have substantially greater and less precise pooled effect sizes compared to those rated as “fair” or “good” using the USPSTF system (Figure 1).

In general, the USPSTF system was also found to be more conservative, with six of the 20 studies assigned a lower overall quality rating (Table 1). One possible reason accounting for this finding is that the USPSTF system considers a number of potentially invalidating methodological flaws in its assessment. The Downs and Black scale on the other hand assigns each question a single point (except in one case where a single question may be awarded two points). As a result, a study can contain a potentially serious flaw, and still be rated as “fair” or “good” quality.

Since the USPSTF system gives equal weighting to external validity, this might have accounted for the differences. However, the reasons for studies being rated as “poor” generally related to issues of internal validity, such as inadequate allocation concealment in randomized controlled trials, or possible selection bias occurring due to unequal distribution of primary outcomes at baseline. Schulz and co-authors [24] suggest that allocation concealment is the element of quality that is associated with the greatest risk of bias. While the greater effect sizes compared to those rated as “fair” or “good” was based on only three “poor” quality studies, others have reported similar findings using larger numbers of included studies [24–27].

The influence of other quality factors on effect size are less certain [5]; and various issues apart from methodological quality may contribute to inexact treatment effect sizes, including heterogeneity of study interventions or sample populations [28, 29]. Although we included studies which were generally homogenous in terms of intervention type and sample population, it is uncertain whether differences in methodological quality alone would account for the variations in treatment effect observed in those studies rated as “poor”.

### Strengths and limitations

These results should be considered with a degree of caution given the relatively small number of included studies, and assessing a larger number of heterogeneous studies would be required to provide more certain evidence in support of these findings. Despite this, the study provides further support for the contention that numerical summary scores should not be used for the assessment of methodological quality, or for determining cut-off criteria for study inclusion. In practical terms, within the specific example of a single systematic review [15], a commonly used numerical summary scale failed to identify the small number of included studies which contained important sources of potential bias according to the domain based system.

While we found a good level of reliability between independent assessments for both tools it is acknowledged that this could be due to the pilot phase used to standardize scoring methods, and the relatively small number of studies [30, 31]. The conversion of domain based USPSTF ratings to a numerical value for reliability assessment is also a limitation; however this was to allow for comparison to be made with the Downs and Black scale and since it would provide a more robust and sensitive measure than comparing ratings of “poor” “fair” or “good”. A further limitation is that there is no gold standard with which quality assessment tools can be compared. The study also did include a qualitative assessment of utility.

We selected the Downs and Black scale as it is one of the most widely used and well validated tools for assessment of both randomized and non-randomized studies [18]. However, in comparison to the USPSTF system, a number of limitations associated with its use were identified. In particular, the ability of the Downs and Black scale to differentiate studies containing potential sources of bias was limited in comparison to the USPSTF system.

### Recommendations

Summary quality scales combine information on several methodological features in a single numerical value, whereas component or domain-based approaches examine key dimensions or outcomes individually [6, 12–14]. The use of summary scores from numerical rating scales for assessment of methodological quality has been called into question [4, 8, 32]. One issue is that they frequently incorporate items such as quality of reporting, ethical issues or statistical analysis techniques which are not directly related to quality or to potential bias [4]. This is an important distinction, since the inclusion of such items may be misleading and a study containing methodological bias, but which is well reported, can potentially still be rated as high quality. In particular, the practice of using numerical scores to identify trials of apparent low or high quality in a systematic review is not recommended [32].

Analysis of individual components of quality may overcome many of the shortcomings of composite scores. The component approach takes into account the importance of individual quality domains, and that the direction of potential bias varies between the contexts in which studies are performed [33]. Decisions relating to assessment of methodological quality when using domain-based rating systems are therefore dependent upon the particular research area under consideration, since important components relating to bias are not universal. The use of a standard set of quality components across all clinical areas is not recommended [5] and more specific guidance may be required when using these types of assessment tool [33, 34]. Review authors should therefore remain cautious when using a domain based system to assess methodological quality and formulate guideline recommendations.

## Conclusions

Here we evaluated a domain-based rating system and demonstrated its ability to successfully differentiate studies associated with potentially exaggerated treatment effects. Domain-based rating systems provide a structured framework by which studies can be assessed in a qualitative manner, allowing for the identification of potential sources of bias, firstly within the individual studies, but also in the context of the available body of evidence under review. This is important as quality of evidence can vary across outcomes reported in the same study, and some outcomes may be more prone to bias than others. For example, bias due to lack of allocation concealment may be more likely for subjective outcomes, such as quality of life [29]. How to account for any potential bias in the analysis remains in question, but the current Cochrane guidelines [11] recommend examining studies containing potential methodological bias as a separate sub-category in a sensitivity analysis.

## Additional files

Additional file 1:
**Table S1.** Comparison of different criteria included in the Downs and Black scale and USPSTF system quality assessment tools. Additional file 2:
**Table S2.** References to studies included in the comparison of quality assessment methods.

## Abbreviations

USPSTF: United States Preventative Services Task Force
ICC: intraclass correlation coefficient
SMD: standardized mean difference
PRISMA: preferred reporting items for systematic reviews and meta-analyses
95% CI: 95% confidence interval.

## References

[1] Liberati A, Altman DG, Tetzlaff J, Mulrow C, Gøtzsche PC, Ioannidis JP (2009). The PRISMA statement for reporting systematic reviews and meta-analyses of studies that evaluate health care interventions: explanation and elaboration. Ann Intern Med.

[2] Kirkham JJ, Gargon E, Clarke M, Williamson PR (2013). Can a core outcome set improve the quality of systematic reviews?—a survey of the Co-ordinating Editors of Cochrane Review Groups. Trials.

[3] Kirkham JJ, Dwan KM, Altman DG, Gamble C, Dodd S, Smyth R (2010). The impact of outcome reporting bias in randomised controlled trials on a cohort of systematic reviews. BMJ.

[4] Hartling L, Ospina M, Liang Y, Dryden DM, Hooton N, Krebs Seida J (2009). Risk of bias versus quality assessment of randomised controlled trials: cross sectional study. BMJ.

[5] Wood L, Egger M, Gluud LL, Schulz KF, Jüni P, Altman DG (2008). Empirical evidence of bias in treatment effect estimates in controlled trials with different interventions and outcomes: meta-epidemiological study. BMJ.

[6] Guyatt GH, Oxman AD, Vist G, Kunz R, Brozek J, Alonso-Coello P (2011). GRADE guidelines: 4. Rating the quality of evidence—study limitations (risk of bias). J Clin Epidemiol.

[7] Colle F, Rannou F, Revel M, Fermanian J, Poiraudeau S (2002). Impact of quality scales on levels of evidence inferred from a systematic review of exercise therapy and low back pain. Arch Phys Med Rehabil.

[8] Gagnier JJ, Kellam PJ (2013). Reporting and methodological quality of systematic reviews in the orthopaedic literature. J Bone Joint Surg Am.

[9] Jüni P, Tallon D, Egger M (2000) ‘Garbage in–garbage out’? Assessment of the quality of controlled trials in meta-analyses published in leading journals. In: Proceedings of the 3rd symposium on systematic reviews: beyond the basics. St Catherine’s College, Oxford. Centre for Statistics in Medicine, Oxford, p 19

[10] Herbison P, Hay-Smith J, Gillespie WJ (2006). Adjustment of meta-analyses on the basis of quality scores should be abandoned. J Clin Epidemiol.

[11] Higgins JPT, Green S (eds) (2009) Cochrane handbook for systematic reviews of interventions version 5.0.2 [updated September 2009]. The Cochrane Collaboration

[12] Harris RP, Helfand M, Woolf SH, Lohr KN, Mulrow CD, Teutsch SM (2001). Current methods of the US Preventive Services Task Force: a review of the process. Am J Prev Med.

[13] Sawaya GF, Guirguis-Blake J, LeFevre M, Harris R, Petitti D (2007). Force USPST: update on the methods of the US Preventive Services Task Force: estimating certainty and magnitude of net benefit. Ann Intern Med.

[14] Atkins D, Eccles M, Flottorp S, Guyatt GH, Henry D, Hill S (2004). Systems for grading the quality of evidence and the strength of recommendations I: critical appraisal of existing approaches The GRADE Working Group. BMC Health Serv Res.

[15] O’Connor SR, Tully MA, Ryan B, Bleakley CM, Baxter GD, Bradley JM et al (2014) Walking exercise for chronic musculoskeletal pain: systematic review and meta-analysis. Arch Phys Med Rehabil. S0003-9993(14)01314-8 [pii] [Epub ahead of print] 10.1016/j.apmr.2014.12.003 25529265

[16] Downs SH, Black N (1998). The feasibility of creating a checklist for the assessment of the methodological quality both of randomised and non-randomised studies of health care interventions. J Epidemiol Community Health.

[17] Deeks JJ, Dinnes J, D’Amico R, Sowden AJ, Sakarovitch C, Song F et al (2003) Evaluating non-randomised intervention studies. Health Technol Assess 7(27):iii–x, 1–173 10.3310/hta7270 14499048

[18] Richmond SA, Fukuchi RK, Ezzat A, Schneider K, Schneider G, Emery CA (2013). Are joint injury, sport activity, physical activity, obesity, or occupational activities predictors for osteoarthritis? A systematic review. J Orthop Sports Phys Ther.

[19] Morton S, Barton CJ, Rice S, Morrissey D (2014). Risk factors and successful interventions for cricket-related low back pain: a systematic review. Br J Sports Med.

[20] Simic M, Hinman RS, Wrigley TV, Bennell KL, Hunt MA (2011). Gait modification strategies for altering medial knee joint load: a systematic review. Arthritis Care Res (Hoboken).

[21] Moher D, Liberati A, Tetzlaff J, Altman DG, PRISMA Group (2009). Preferred reporting items for systematic reviews and meta-analyses: the PRISMA statement. Ann Intern Med.

[22] McDowell I (2006) Chapter 2: the theoretical and technical foundations of health measurements. In: Measuring health, 3rd edn. Oxford University Press, Oxford, pp 10–54

[23] The Cochrane Collaboration (2008). Review Manager (RevMan) [Computer program]. Version 50.

[24] Schulz KF, Chalmers I, Hayes RJ, Altman DG (1995). Empirical evidence of bias. Dimensions of methodological quality associated with estimates of treatment effects in controlled trials. JAMA.

[25] Siersma V, Als-Nielsen B, Chen W, Hilden J, Gluud LL, Gluud C (2007). Multivariable modelling for meta-epidemiological assessment of the association between trial quality and treatment effects estimated in randomized clinical trials. Stat Med.

[26] Moja LP, Telaro E, D'Amico R, Moschetti I, Coe L, Liberati A (2005). Assessment of methodological quality of primary studies by systematic reviews: results of the metaquality cross sectional study. BMJ.

[27] Odgaard-Jensen J, Vist GE, Timmer A, Kunz R, Akl EA, Schünemann H et al (2011) Randomisation to protect against selection bias in healthcare trials. Cochrane Database Syst Rev (4):MR000012 10.1002/14651858.MR000012.pub3 PMC7150228 21491415

[28] Savović J, Jones HE, Altman DG, Harris RJ, Jüni P, Pildal J (2012). Influence of reported study design characteristics on intervention effect estimates from randomized, controlled trials. Ann Intern Med.

[29] Hartling L, Milne A, Hamm MP, Vandermeer B, Ansari M, Tsertsvadze A (2013). Testing the Newcastle Ottawa Scale showed low reliability between individual reviewers. J Clin Epidemiol.

[30] Oremus M, Oremus C, Hall GB, McKinnon MC, Team EaCSR (2012). Inter-rater and test–retest reliability of quality assessments by novice student raters using the Jadad and Newcastle-Ottawa Scales. BMJ Open.

[31] Aubut JA, Marshall S, Bayley M, Teasell RW (2013). A comparison of the PEDro and Downs and Black quality assessment tools using the acquired brain injury intervention literature. NeuroRehabilitation.

[32] Greenland S (1994). Quality scores are useless and potentially misleading. Am J Epidemiol.

[33] Balk EM, Bonis PA, Moskowitz H, Schmid CH, Ioannidis JP, Wang C (2002). Correlation of quality measures with estimates of treatment effect in meta-analyses of randomized controlled trials. JAMA.

[34] Armijo-Olivo S, Stiles CR, Hagen NA, Biondo PD, Cummings GG (2012). Assessment of study quality for systematic reviews: a comparison of the Cochrane Collaboration Risk of Bias Tool and the Effective Public Health Practice Project Quality Assessment Tool: methodological research. J Eval Clin Pract.

