# Supplementary material for: Failure of a numerical quality assessment scale to identify potential risk of bias in a systematic review: a comparison study
Source: BMC Res Notes. 2015 Jun 6;8:224. doi: 10.1186/s13104-015-1181-1 (PMC4467625; doi:10.1186/s13104-015-1181-1)
Supplement: Additional file 1: — Table S1. Comparison of different criteria included in the Downs and Black scale and USPSTF system quality assessment tools. [file 13104_2015_1181_MOESM1_ESM.docx]

### Additional file 1 - Comparison of different criteria included in the Downs and Black scale and USPSTF system quality assessment tools.

### IVQ = Internal validity related question; EVQ = External validity related question

| **Downs and Black** | **United States Preventative Services Task Force** |
| --- | --- |
| ***Reporting*** | |
| ***1.*** *Is the hypothesis/aim/objective of the study clearly described?* | *No direct comparison* |
| ***2.*** *Are the main outcomes to be measured clearly described in the Introduction or Methods section?* | *No direct comparison* |
| ***3.*** *Are the characteristics of the patients included in the study clearly described?* | ***(EVQ2)*** *Similarities of the populations studied (demographics, ethnicity, gender, clinical presentation)* |
| ***4.*** *Are the interventions of interest clearly described?* | ***(IVQ5)*** *Clear definition of interventions* |
| ***5.*** *Are the distributions of principal confounders in each group of subjects to be compared clearly described?* | ***(IVQ1)*** *Initial assembly of comparable groups: For RCTs: adequate randomization including concealment and whether potential confounders were distributed equally among groups. For cohort studies: consideration of potential confounders with either restriction or measurement for adjustment in the analysis; consideration of inception cohorts* |
| ***6.*** *Are the main findings of the study clearly described?* | *No direct comparison* |
| ***7.*** *Does the study provide estimates of the random variability in the data for the main outcomes?* | *No direct comparison* |
| ***8.*** *Have all important adverse events that may be a consequence of the intervention been reported?* | *Considered at later stage of assessment using US system *** |
| ***9.*** *Have the characteristics of patients lost to follow-up been described?* | ***(IVQ2)*** *Maintenance of comparable groups (includes attrition, crossovers, adherence, contamination* |
| ***10.*** *Have actual probability values been reported (e.g. 0.035 rather than <0.05) for the main outcomes except where the probability value is less than 0.001?* | *No direct comparison* |
| ***External validity*** | |
| *No direct comparison* | ***(EVQ1)*** *biologic plausibility* |
| ***11.*** *Were the subjects asked to participate in the study representative of the entire population from which they were recruited?* | ***(EVQ2)*** *similarities of the populations studied and primary care patients (in terms of risk factor profile, demographics, ethnicity, gender, clinical presentation, and similar factors)* |
| ***12.*** *Were those subjects who were prepared to participate representative of the entire population from which they were recruited?* |  |
| *No direct comparison* | ***(EVQ3)*** *similarities of the test or intervention studied to those that would be routinely available or feasible in typical practice* |
| ***13.*** *Were the staff, places, and facilities where the patients were treated, representative of the treatment the majority of patients receive?* | ***(EVQ4)*** *clinical or social environmental circumstances in the studies that could modify the results from those expected in a primary care setting* |
| ***Internal validity – bias*** | |
| ***14.*** *Was an attempt made to blind study subjects to the intervention they have received ?* | ***(EVQ4)*** *Measurements: equal, reliable, and valid (includes masking of outcome assessment)* |
| ***15.*** *Was an attempt made to blind those measuring the main outcomes of the intervention?* |  |
| ***16.*** *If any of the results of the study were based on “data dredging”, was this made clear?* | *No direct comparison* |
| ***17.*** *In trials and cohort studies, do the analyses adjust for different lengths of follow-up of patients, or in case-control studies, is the time period between the intervention and outcome the same for cases and controls ?* | *No direct comparison* |
| ***18.*** *Were the statistical tests used to assess the main outcomes appropriate?* | *No direct comparison* |
| ***19.*** *Was compliance with the intervention/s reliable?* | ***(IVQ2)*** *Maintenance of comparable groups (includes attrition, crossovers, adherence, contamination)* |
| ***20.*** *Were the main outcome measures used accurate (valid and reliable)?* | ***(IVQ4)*** *Measurements: equal, reliable, and valid (includes masking of outcome assessment)* ***(IVQ6)*** *All important outcomes considered* |
| ***21.*** *Were the patients in different intervention groups (trials and cohort studies) or were the cases and controls (case-control studies) recruited from the same population?* | *No direct comparison* |
| ***Internal validity - confounding (selection bias)*** | |
| ***22****. Were study subjects in different intervention groups (trials and cohort studies) or were the cases and controls (case-control studies) recruited over the same period of time?* | *No direct comparison* |
| ***23.*** *Were study subjects randomised to intervention groups?* | ***(IVQ1)*** *Initial assembly of comparable groups: For RCTs: adequate randomization including concealment and whether potential confounders were distributed equally among groups. For cohort studies: consideration of potential confounders with either restriction or measurement for adjustment in the analysis; consideration of inception cohorts* |
| ***24.*** *Was the randomised intervention assignment concealed from both patients and health care staff until recruitment was complete and irrevocable?* | ***(IVQ1)*** *Initial assembly of comparable groups: For RCTs: adequate randomization including concealment and whether potential confounders were distributed equally among groups. For cohort studies: consideration of potential confounders with either restriction or measurement for adjustment in the analysis; consideration of inception cohorts.*  ***(IVQ4)*** *Measurements: equal, reliable, and valid (includes masking of outcome assessment)* |
| ***25.*** *Was there adequate adjustment for confounding in the analyses from which the main findings were drawn?* | ***(IVQ7)*** *Analysis: adjustment for potential confounders for cohort studies, or intention-to-treat analysis for RCTs* |
| ***26.*** *Were losses of patients to follow-up taken into account?* | ***(IVQ2)*** *Maintenance of comparable groups (includes attrition, crossovers, adherence, contamination)*  ***(IVQ3)*** *Important differential loss to follow-up or overall high loss to follow-up* |
| ***Power*** | |
| ***27.*** *Did the study have sufficient power to detect a clinically important effect where the probability value for a difference being due to chance is less than 5%?* | *No direct comparison* |

** The USPSTF system consists of a number of strata of which quality assessment is the first step. Adverse events are considered as part of a later stage where the reported and potential harms associated with the intervention in question are considered.
