# Supplementary material for: Failure of a numerical quality assessment scale to identify potential risk of bias in a systematic review: a comparison study
Source: BMC Res Notes. 2015 Jun 6;8:224. doi: 10.1186/s13104-015-1181-1 (PMC4467625; doi:10.1186/s13104-015-1181-1)
Supplement: Additional file 2: — Table S2. References to studies included in the comparison of quality assessment methods. [file 13104_2015_1181_MOESM2_ESM.docx]

**Additional file 2 - References to studies included in the comparison of quality assessment methods**

| Bautch JC, Malone DG, Vailas AC. Effects of exercise on knee joints with osteoarthritis: a pilot study of biologic markers. Arthritis Care Res. 1997;10:48-55. |
| --- |
| Bautch JC, Clayton MK, Chu Q, et al. Synovial fluid chondroitin sulphate epitopes 3B3 and 7D4, and glycosaminoglycan in human knee osteoarthritis after exercise. Ann Rheum Dis. 2000;59:887-91. |
| Bircan C, Karasel SA, Akgün B, et al. Effects of muscle strengthening versus aerobic exercise program in fibromyalgia. Rheumatol Int. 2008;28:527-32. |
| Dias RC, Dias JM, Ramos LR. Impact of an exercise and walking protocol on quality of life for elderly people with OA of the knee. Physiother Res Int. 2003;8:121-130. |
| Ettinger WH, Burns R, Messier SP, et al. A randomized trial comparing aerobic exercise and resistance exercise with a health education program in older adults with knee osteoarthritis. The Fitness Arthritis and Seniors Trial (FAST). JAMA. 1997;277:25-31. |
| Evcik D and Sonel B. Effectiveness of a home-based exercise therapy and walking program on osteoarthritis of the knee. Rheumatol Int. 2002;22:103-6. |
| Ferrell BA, Josephson KR, Pollan AM, et al. A randomized trial of walking versus physical methods for chronic pain management. Aging. 1997;9:99-105. |
| Holtgrefe K, McCloy C, Rome L. Changes associated with a quota-based approach on a walking program for individuals with fibromyalgia. J Orthop Sports Phys Ther. 2007;37:717-24. |
| Koldas Doğan S, Sonel Tur B, Kurtais Y et al. Comparison of three different approaches in the treatment of chronic low back pain. Clin Rheumatol. 2008;27:873-881. |
| Kovar PA, Allegrante JP, MacKenzie CR, et al. Supervised fitness walking in patients with osteoarthritis of the knee. A randomized, controlled trial. Ann Intern Med. 1992;116:529-534. |
| Lemstra M and Olszynski WP. The effectiveness of multidisciplinary rehabilitation in the treatment of fibromyalgia: a randomized controlled trial. Clin J Pain. 2005;21:166-74. |
| Martin L, Nutting A, MacIntosh BR, et al. An exercise program in the treatment of fibromyalgia. J Rheumatol. 1996;23:1050-3. |
| Messier SP, Loeser RF, Miller GD, et al. Exercise and Dietary Weight Loss in Overweight and Obese Older Adults With Knee Osteoarthritis: The Arthritis, Diet, and Activity Promotion Trial. Arthritis Rheum. 2004;50:1501-10. |
| Meyer BB and Lemley KJ. Utilizing exercise to affect the symptomology of fibromyalgia: a pilot study. Med Sci Sports Exerc. 2000;32:1691-7. |
| Miller GD, Nicklas BJ, Davis C, et al. Intensive Weight Loss Program Improves Physical Function in Older Obese Adults with Knee Osteoarthritis. Obesity. 2006;14:1219-30. |
| Nichols DS and Glenn TM. Effects of aerobic exercise on pain perception, affect, and level of disability in individuals with fibromyalgia. Phys Ther. 1994;74:327-32. |
| Rasmussen-Barr E, Ang B, Arvidsson I, et al. Graded Exercise for Recurrent Low-Back Pain. A Randomized, Controlled Trial With 6-, 12-, and 36-Month Follow-ups. Spine. 2009;34:221-228. |
| Rooks DS, Gautam S, Romeling M, et al. Group exercise, education, and combination self-management in women with fibromyalgia: a randomized trial. Arch Intern Med. 2007;12;167:2192-200. |
| Talbot LA, Gaines JM, Huynh TN, et al. A Home-Based Pedometer-Driven Walking Program to Increase Physical Activity in Older Adults with Osteoarthritis of the Knee: A Preliminary Study. J Am Geriatr Soc. 2003;51:387-92. |
| Valim V, Oliveira L, Suda A, et al. Aerobic fitness effects in fibromyalgia. J Rheumatol. 2003;30:1060-9. |
